# The Antiangiogenic Effect and Ocular Pharmacology of Novel Modified Nonsteroidal Anti-Inflammatory Drugs in the Treatment of Oxygen-Induced Retinopathy

**DOI:** 10.1089/jop.2022.0113

**Published:** 2023-05-10

**Authors:** Wei Huang, Liqun Huang, Ziyi Wen, Robert A. Honkanen, Basil Rigas

**Affiliations:** ^1^ Department of Ophthalmology, Population and Preventive Medicine, Stony Brook University, Stony Brook, New York, USA.; ^2^ Medicon Pharmaceuticals, Inc., Setauket, New York, USA.; ^3^ Department of Family, Population and Preventive Medicine, Stony Brook University, Stony Brook, New York, USA.

**Keywords:** oxygen-induced retinopathy, OXT-328, Q-922, CL-717, abnormal vessel growth, retinal angiogenesis

## Abstract

**Purpose:**

To evaluate the hypothesis that 3 novel compounds, OXT-328, Q-922, and CL-717 show efficacy in the treatment of oxygen-induced retinopathy (OIR) and whether or not their route of administration is intravitreal, topical, or systemic.

**Methods:**

The OIR mouse model, characterized by an avascular area (AVA) and a neovascular area (NVA) of the retina, was used to study retinopathy of prematurity and other retinal diseases characterized by abnormal vessel growth. We measured the effect of our compounds on both the AVA and NVA in whole mounts of mouse retinal tissue. We also evaluated their ability to prevent new vessel formation in chicken chorioallantoic membranes (CAMs). Finally, we measured the *in vitro* uptake and biodistribution of topically applied CL-717 in human eye explants.

**Results:**

In mice with OIR, compared to controls, a single intravitreal administration of Q-922 or OXT-328 significantly reduced both AVA and NVA. CL-717 administered as eye drops over 5 days also reduced AVA and NVA, whereas OXT-328 eye drops had no effect. Q-922 given intraperitoneal (150 mg/kg/day × 5 days) reduced AVA and NVA. Remarkably, explanted human eyes bathed in CL-717 show rapid uptake and biodistribution in ocular tissues. In the chicken CAM model, all 3 compounds reduced the formation of new blood vessels by about one-third. No side effect in mice was observed, except for mild ocular surface irritation with Q-922.

**Conclusions:**

Systemic administration of Q-922 or topical administration of CL-717 holds particular promise for a simplified treatment of proliferative retinopathies without the necessity of intravitreal injections.

## Introduction

Abnormal vessel growth is the hallmark of many retinal diseases, including the retinopathy of prematurity (ROP), wet age-related macular degeneration, and proliferative diabetic retinopathy (DR). A mouse model of oxygen-induced retinopathy (OIR) is in extensive use to study retinal neovascularization and vascular leakage in normoglycemic animals. The underlying seminal observation is that when newborn mice exposed to hyperoxic conditions postnatally (days 7–12) are returned to normal air, the retina experiences a temporary state of relative hypoxia. This hypoxia is the stimulus for neovascularization^1,2^ and is associated with the release of angiogenic factors.^3,4^ The OIR model recapitulates key features of ROP in newborns^5^ and is characterized by retinal neovascularization, growth of nonperfused vessels, and microaneurysms occurring within 5 days postexposure to room air.^6^

The OIR model offers several experimental advantages such as reproducibility and ease of visualization and quantification of the neovascularization. These advantages make the OIR model popular for the study of mechanisms of, and potential therapeutics for ischemic proliferative retinopathies, including ROP and DR.^7^

Others have reported that nonsteroidal anti-inflammatory drugs (NSAIDs) like aspirin and sulindac have antiproliferative as well as antiangiogenic properties.^8–11^ We synthesized 3 novel NSAIDs (OXT-328, Q-922, and CL-717) and hypothesized that they have the ability to prevent and/or suppress the neovascularization, which occurs in the OIR model. We have previously reported that various phospho-NSAIDs, including our lead compound phospho-sulindac (OXT-328), demonstrate antiproliferative properties in preclinical models of cancer, including cancers of the colon, skin, pancreas, and lung.^12–15^

A significant structural feature of OXT-328 is its carboxylic ester bond linking the conventional sulindac moiety to the butyl spacer. While this bond is important for its pharmacological activity, it also renders the molecule susceptible to hydrolysis by carboxylesterases.^16^ In animals such as mice that express these enzymes abundantly,^17^ the systemic administration of OXT-328 leads to its rapid degradation with only trace levels found in ocular tissues.^18^ A new series of compounds, including the 2 reported here, Q-922 and CL-717, overcomes this limitation since the carboxylic ester is replaced with a nonhydrolyzable bond, making them resistant to esterases and permitting their administration systemically or as eye drops.

Our overarching goal was to identify compounds that combine efficacy with a mode of administration that obviates the need for intravitreal injections. Using eye drops represents one of the simplest ways of administering ocular drugs with some allowance for the drug's lipophilicity and the galenic form of eye drops when considering transport across corneal and conjunctival barriers Compared to anterior chamber targeting, the delivery of drugs to the retina by eye drops may be more difficult. However, there are reports in rabbits, rats, and mice that antiproliferative doses of small molecular weight drugs can be delivered to the retina by eye drops.^19,20^ In addition to immediate tissue targeting, eye drops enhance their safety because they use very small amounts of drug, often preventing drugs from reaching the systemic circulation at pharmacological meaningful concentrations.

Systemic administration of drugs is in some cases easier than eye drops such as with orally administered drugs. However, this increases the possibility of side effects due to a wider biodistribution and larger doses. While the vitreous provides a storage tissue from which therapeutic agents can reach the retina, their residence time there is limited; for example, peptides have intravitreal half-lives of about 1 week.^21^ Thus, to be efficacious, such agents require frequent injection into the vitreous. This creates a significant drawback to their continued use for the treatment for proliferative retinopathies.

Based on these considerations, we evaluated the effect of our 3 compounds on OIR by administering them to the murine eyes through 3 different routes, that is, eye drops, intravitreal injection, and systemic administration. In addition, we studied the pharmacokinetics and biodistribution of these compounds in adult murine ocular tissues and human eye explants. The effect of these modified NSAIDs was also determined in another model of angiogenesis, the *ex ovo* chicken chorioallantoic membrane (CAM) assay.

Our data demonstrate that these novel compounds have significant efficacy in both models of angiogenesis. Intravitreal injection of OXT-328 decreased angiogenesis in the mouse model of OIR, but not in eye drop form. Remarkably, one of them, CL-717, displayed strong efficacy when given as eye drops and a second, Q-922, displayed similar efficacy when administered intraperitoneally. Taken together, our findings raise the possibility that these novel compounds could become important therapeutic agents for the treatment of proliferative retinopathies and related diseases.

## Methods

### Reagents

Alexa Fluor 488 conjugated isolectin-B4 was purchased from Vector Labs, Burlingame, CA, and PermaFluorTM aqueous mounting medium from Thermo Fisher Scientific, Waltham, MA. All other chemicals and reagents were purchased from Sigma-Aldrich Corp., St. Louis, MO, and were of HPLC grade or the highest grade available.

### Test compounds

OXT-328 (phospho-sulindac) was a gift from Medicon Pharmaceuticals, Inc., Setauket, NY, USA. The Supplemental Data File S1 presents the chemical structures of OXT-328, Q-922 and CL-717 (Supplementary Fig. S1) as well as the details of the synthesis of both Q-922, a heavily modified form of aspirin (Supplementary Figs. S2 and S4), and CL-717, a modified form of sulindac (Supplementary Figs. S3 and S4).

### Animals

All animal studies were prospectively approved by the Stony Brook University Institutional Animal Care and Use Committee (Protocol#1151093) and performed in accordance with the Association of Research in Vision and Ophthalmology Statement for the Use of Animals in Ophthalmic and Vision Research. Adult mice were euthanized with inhaled CO_2_ and neonates were euthanized by cervical dislocation due to their greater tolerance to CO_2_. In the OIR model, mice were euthanized on postnatal day 17, while in other experiments, mice were euthanized at hourly intervals, up to 18 h after administration of test drugs. To minimize the discomfort of intravitreal injections, mice were anesthetized with an intraperitoneal injection of ketamine (79.5 mg/kg) and xylazine (9.1 mg/kg).

C57BL/6 mice with or without pregnancy (Subline J, specific pathogen-free class) were purchased from Jackson Laboratory, Bar Harbor, ME, and housed in room air (21% oxygen) at 25°C and a 12-h light/12-h dark cycle, with free access to food and water. Animal experiments were designed to provide intralitter control and equal size groups. We used randomization and blinded analysis. After delivery, pups from 1 litter were randomized into groups, and the number of mice in each group was *n* ≥ 6.

### Human eyes

Human eyes were obtained through the Lions Eye Bank for Long Island, Valley Stream, NY. These human tissues are cadaveric in origin and their use does not require IRB approval (see 45 CFR §46.102). The donor's identity was protected by de-identifying each sample. Following harvesting, they were transported on ice and delivered to the laboratory for study within 2 h after enucleation.

### OIR mouse model

Pup litters together with their mothers were exposed to 75% oxygen from postnatal day 7 (P7) to P12 and returned to room air from P12 to P17. Table 1 shows their treatment schedule, drug doses, and routes of administration.

**Table 1.** Drug Administration to Mice with Oxygen-Induced Retinopathy

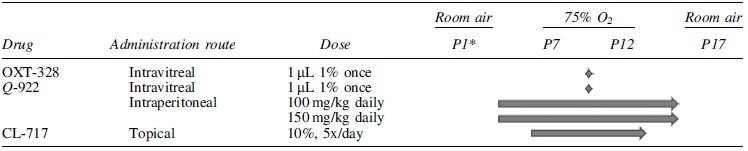

^*^ P1–P17: Postnatal days.

### Retinal flat mounts

For retinal flat mounts,^22^ on P17, the eyeballs of the mice were enucleated and fixed in 4% paraformaldehyde for 0.5 h. Once the eyeball was fixed, its posterior segment was excised and fixed further in the same solution for 1 h, after which the retina was dissected and fixed again in 4% paraformaldehyde overnight at 4°C. Following this, the retina was washed sequentially as follows: thrice in phosphate-buffered saline (PBS) with 0.1% Tween-20 for 10 min each; in 50% methanol for 10 min; in 100% methanol for 10 min; and thrice in PBS with 0.1% Tween-20 for 10 min each. The retina was then blocked with 1% bovine serum albumin in 0.5% Triton X-100 in PBS for 30 min; incubated with Alexa Fluor 488 conjugated isolectin-B4 overnight at 4°C; washed 4 times in 0.1% Tween-20 in PBS; mounted on a glass slide; and covered with PermaFluorTM aqueous mounting medium.

### Retinal image analysis

Retinal flat mounts were imaged with an optical microscope at 10 × magnification, and the avascular area (AVA) and neovascular area (NVA) were delineated with ImageJ, a public domain Java image processing and analysis program. The software provides the user with the ability to determine total image surface area, AVA and NVA. Noncontinguous regions of interest such as AVA and NVA could be outlined and the areas could be summed. Results are expressed as a percent of the total surface area in dimensionless units. The person measuring the AVA and NVA was blinded as to the treatment received by that animal.

### Experimental design

Table 1 summarizes the routes of administration of each study drug (OXT-328, Q-922, CL-717). From previous studies, we knew that our lead compound OXT-328 was subject to rapid hydrolysis by carboxylesterases. Thus, to demonstrate its efficacy in the mouse model, OXT-328 was administered intravitreally but not i.p. or as eye drops.

The purpose of the synthesis of 2 other novel NSAIDs (Q-922 and CL-717) was to create test drugs with the same efficacy of OXT-328 in inhibiting abnormal vessel growth, but not subjected to hydrolysis by carboxylesterases. Thus, we studied the effects of eye drops compounded with Q-922 and CL-717. As described below, studies with eye drops containing Q-922 were discontinued; however, the effect of Q-922 when administered as an intravitreal or intraperitoneal injection is reported below. The effect of CL-717 was only studied when administered in eye drop form.

Also, it should be noted that dosing when study drug was administered as an eye drop or as an intravitreal injection is reported as the volume of the solution containing % weight in grams of test drug divided by weight of solutions. Only i.p. injections are expressed as mg/kg body weight. The reason for expressing the dose in this manner is because the test neonates vary in body weight from birth until day P17 from 0.5 to 10 g.

### Drug administration and dosage

#### Eye drops

We used a 10% solution of the water-soluble CL-717 in ddH_2_O with its pH adjusted to 5.0 ± 0.2 with NaOH, and its osmolality to 300 ± 20 mOsm/kg H_2_O with 18% NaCl. This solution of CL-717 was applied to the ocular surface as 5 μL eye drops, as indicated in Table 1. To formulate a 7.15% solution of Q-922 for topical delivery, 71.5 mg of Q-922 was dissolved in 800 μL of a 5% Tween 80/95% normal saline solution, pH 7.0. Additions of 150 μL of 15% solution of polyethylene glycol and 50 μL of 5% solution of Kolliphor EL were made to the Q-922 solution.

The pH was adjusted to 6.8 and the final volume was 1 mL. Q-922 similar to CL-717 was applied to the ocular surface as 5 μL eye drops when P12 mice were returned to room air. To prepare OXT-328 eye drops, vitamin E TPGS (D-α-tocopheryl polyethylene glycol 1000 succinate) and polyquaternium-1 were dissolved in purified water. OXT-328 (3.5%) was added to this solution, and stirred at 70°C for 30 min. This solution was then centrifuged at 13,200 rpm for 10 min and the supernatant was collected. Mannitol and boric acid were added to the harvested supernatant. After pH adjustment to 6.7 ± 0.2 using NaOH, purified water was added to the final volume. The resulting solution was sterilized by filtration through a 0.22 μm filter.

For intravitreal injections, Q-922 and OXT-328 were formulated as follows: vitamin E TPGS and polyquaternium-1 were dissolved in ddH2O and the respective drug was added, and the solution was stirred at 70°C for 30 min. This solution was then centrifuged at 13,200 rpm for 10 min and the supernatant was collected. Mannitol and boric acid were added to the supernatant to final concentrations of 3.18% (w/v) and 1.2% (w/v), respectively. After pH adjustment to 6.7 ± 0.2 with NaOH, purified water was added to the final volume. The resulting solutions were sterilized by filtering through a 0.22 μm filter. One microliter of 1% OXT-328 or Q-922 was injected into the vitreous using a 30G Hamilton syringe, which was deliberately angled to avoid lens injury.

For intraperitoneal administration, Q-922 was dissolved in corn oil.

### Pharmacokinetics and biodistribution in mice and in explanted human eyes

In these studies, we used adult mice, giving us more ocular tissue to work with, even though differences may exist between adult and neonatal tissues. For the topical application of drugs to the ocular surface, 3 eye drops of OXT-328 3.5% or CL-717 10% solution were administered to each eye of the study mice, 5 min apart. For the intravitreal injection of OXT-328, 1 μL of 1% solution was injected once after the mice were anesthetized with an intraperitoneal injection of ketamine 79.5 mg/kg and xylazine 9.1 mg/kg. The pupils were dilated with topical 1% tropicamide. At different time points after the last eye drop, the mice were euthanized and their corneas and retinas were collected quickly, immediately frozen in liquid nitrogen, and stored at −80°C until analyzed. The very small size of these eyes made it difficult to assess its biodistribution to the rest of the globe.

The anterior surface of explanted human eyes was immersed in CL-717 10% solution for 1 or 10 min, followed by washing with ddH_2_O. The eyes were placed in a Petri dish with their anterior part facing up, and the dish was covered and incubated for 1 h at 35.5°C, the normal *in vivo* eye temperature. At that point, eye tissues, including cornea, aqueous humor, iris, ciliary body, retina, and choroid, were dissected quickly, immediately frozen in liquid nitrogen, and stored at −80°C until analyzed.

Tissue drug levels were determined as previously described.^23,24^ Briefly, each tissue sample was weighed and 100–300 μL ddH_2_O was added, depending on tissue weight, and homogenized. Acetonitrile (twice the volume of the homogenate), was added to the homogenate and the mixture was sonicated for 10 min, centrifuged at 13,200 rpm for 15 min, and analyzed by HPLC.

### Ex ovo chicken CAM assay

Fertilized chick eggs were obtained from Charles River Labs (North Franklin, CT) and transferred to a specialized incubator with an automatic rotating device (Grumbach, Lyon Technologies, Inc., Brutgerate GmbH, Germany) for 3 days at 37°C and 70% humidity. After incubation, the eggs were cracked, and the embryo was transferred to a 100 × 25 mm sterilized Petri dish in a laminar flow hood. The eggs were incubated for 7 days and the sterilized filter paper disc containing water, 50 μM of OXT-328, or 50 μM of Q-922, or 100 μM of CL-717 was plated on the yolk sac membrane. The filter papers were treated with each reagent twice per day. Three days later, the angiogenic response was expressed as microvessel density in the CAM area determined under a microscope.^25^

### Statistical analyses

Results, obtained from at least 3 independent experiments, were expressed as mean ± standard error of the mean (SEM), and statistically significant differences between 2 groups were analyzed by Student's *t*-test and one-way analysis of variance followed by the Tukey test for multiple comparisons. *P* < 0.05 was considered statistically significant.

## Results

### The effect of OXT-328 on OIR and its PK/biodistribution

We studied the effect of OXT-328, our lead modified NSAID, on OIR by administering it topically and intravitreally. Systemic administration of OXT-328 was not attempted, because, as we have reported, systemically administered OXT-328 is rapidly hydrolyzed, being essentially undetectable in plasma and many tissues like the eye.^23^

OXT-328 eye drops had no effect on OIR (data not shown). In contrast, when administered intravitreally, OXT-328 had a marked effect on OIR. Specifically, we studied 4 groups of mice: naive; mice exposed to hyperoxia to develop OIR (hyperoxia control group); and those with OIR, which were treated either with vehicle or with OXT-328 1% injected intravitreally once on day P12.

**FIG. 1.**
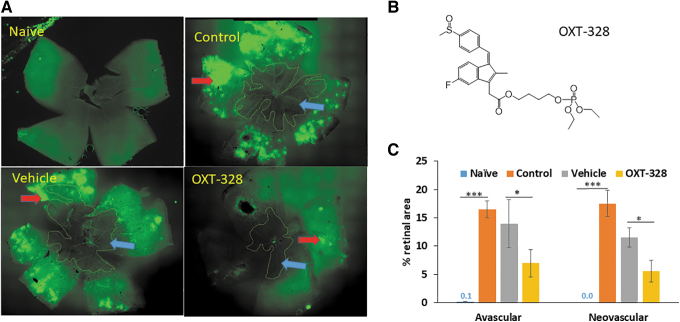
The effect of intravitreally injected OXT-328 on angiogenesis in OIR. **(A)** Representative images of mouse retinal flat mounts stained with the vascular stain isolectin-B4. Mice were treated with vehicle or OXT-328 1% administered as a single 1-μL intravitreal injection on day P12. Retinas were harvested on day P17, as in Methods. *Blue and red arrows*: avascular and neovascular areas, respectively. **(B)** Shows the structure of OXT-328. **(C)** OXT-328 suppressed both the avascular and NVAs of the retina, compared to vehicle-treated and untreated control mice. *n* = 12–16 eyes/group. Values: mean ± SEM. *P < 0.05; ***P < 0.001. NVA, neovascular area; OIR, oxygen-induced retinopathy; SEM, standard error of the mean.

On day P17, 5 days after the hyperoxic exposure, the retinal mounts of the hyperoxia control group when compared to those of naive normoxic mice showed that both the AVA and the NVA were dramatically increased, from 0.1% ± 0.1% to 16.5% ± 1.5% (mean ± SEM for these and all subsequent values); and from 0% ± 0% to 17.5% ± 2.4% of the total retinal area, respectively (Fig. 1A). Treatment of hyperoxic mice with vehicle had no statistically significant effect on retinal vascularity compared to mice from the hyperoxic control group; in the vehicle group, AVA = 13.9% ± 4.2% and NVA = 11.5% ± 1.7%.

In contrast, intravitreal treatment with OXT-328 (chemical structure shown in Fig. 1B) in mice exposed to hyperoxia significantly decreased AVA to 6.9% ± 2.4% and NVA to 5.6% ± 1.9%; *P* < 0.001 for AVA and *P* < 0.03 for NVA when compared to the vehicle group. The changes in AVA and NVA under each experimental condition are shown in Fig. 1C.

**FIG. 2.**
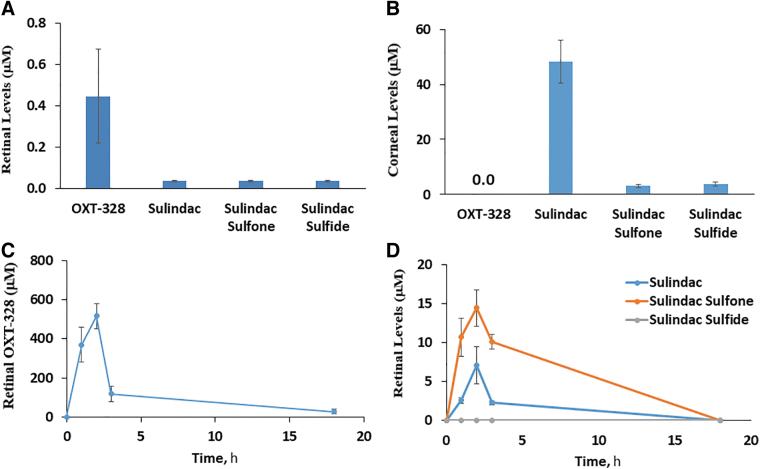
The ocular PK and biodistribution of OXT-328 in adult mice. The levels of OXT-328 and its metabolites in the retina **(A)** and cornea **(B)** 1 h after its administration as eye drops to the mouse ocular surface. **(C)** The retinal levels of OXT-328 after a single 1 μL intravitreal injection of OXT-328 1% into adult mice. C_max_ = 516 ± 64 μM; T_max_ = 2h; and t_1/2_ = 4.8 h. **(D)** Shows the far lower levels of OXT-328 metabolites in the retina of the same mice as in **(C)**, *n* = 6 eyes. Values: mean ± SEM.

The difference in efficacy of the 2 routes of administration, eye drops and intravitreal injection, is explained by their differences in PK/biodistribution. One hour after the administration of OXT-328 as eye drops, its levels in the retina were low (0.4 ± 0.2 μM) and those of its main metabolites 10-fold smaller (Fig. 2A). Of note, OXT-328 was at the same time undetectable in the cornea, where its metabolite sulindac had reached 48.3 ± 7.9 μM, indicating its rapid metabolism to largely inactive metabolites; sulindac sulfide and sulindac sulfone were present at much lower levels (3.1 ± 0.6 and 3.8 ± 0.7 μM, respectively) (Fig. 2B).

These findings are consistent with previous reports on the ocular metabolism of OXT-328 and its hydrolysis by carboxylesterases.^16,24^ In sharp contrast to eye drops, the intravitreal administration of OXT-328 generated robust retinal levels consistent with its pharmacological efficacy: C_max_ = 516.2 ± 64.4 μM, T_max_ = 2 h, and t_1/2_ = 4.8 h (Fig. 2C). Furthermore, its metabolic inactivation was slow, compared to its fate when applied as eye drops to the surface of the eye. The C_max_ of sulindac and sulindac sulfone was 7.1 ± 2.4 μM and 14.5 ± 2.4 μM, respectively (>30-fold lower than OXT-328), whereas sulindac sulfide was undetectable (Fig. 2D).

### The effect of Q-922 on OIR

**FIG. 3.**
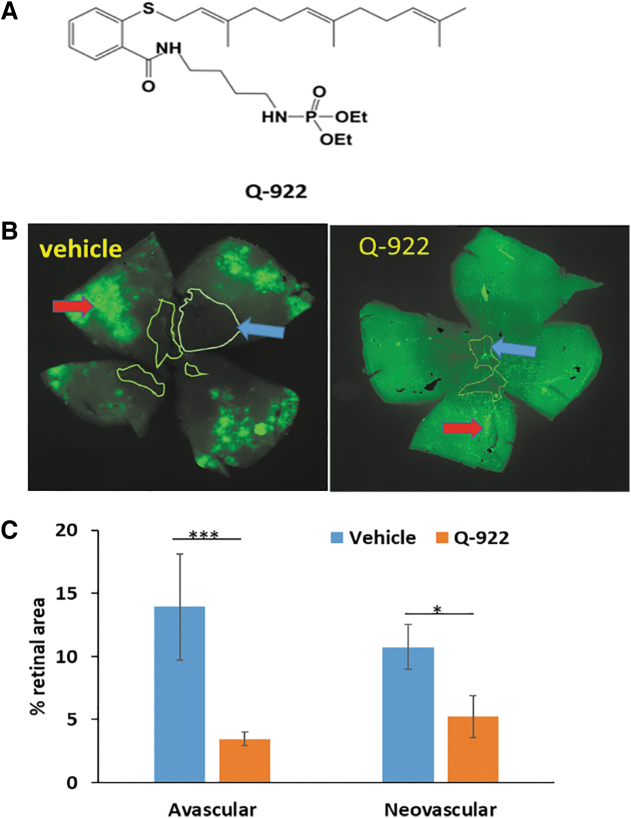
The effect of intravitreally injected Q-922 on angiogenesis in OIR. **(A)** Shows the chemical structure of Q-922, a heavily modified aspirin molecule. **(B)** Representative images of mouse retinal flat mounts stained with the vascular stain isolectin-B4. Mice were treated with vehicle or Q-922 1% administered as a single 1-μL intravitreal injection on day P12. Retinas were harvested on day P17, as in Methods. *Blue and red arrows*: avascular and neovascular areas, respectively. **(C)** Q-922 suppressed both the avascular and NVAs of the retina, compared to vehicle-treated control mice. *n* = 12–16 eyes/group. Values: mean ± SEM. *P < 0.05; ***P < 0.001.

We assessed the effect of Q-922 (chemical structure shown in Fig. 3A) on OIR, employing 3 modes of administration: topical eye drops, intravitreal injection, and intraperitoneal injection (Table 1).

Initially, we investigated the effect of Q-922 eye drops applied to the ocular surface. Q-922, as formulated, caused erythema and mild edema on the ocular surface, although these side effects (not seen with vehicle) subsided within 48 h after treatment was discontinued, and this mode of administration was not evaluated further.

We then studied the efficacy of Q-922 administered as an intravitreal injection (Fig. 3B, C). A single injection of 1 μL of Q-922 1% markedly suppressed both AVA and NVA compared to the vehicle-treated group. Specifically, as shown in Fig. 3C, the AVA in the vehicle group was 13.9% ± 4.2% of the retinal area and in the Q-922-treated group 3.5% ± 0.5% (75% reduction), while the NVA was 10.8% ± 1.8% in the vehicle group and 5.2% ± 1.7% in the Q-922 group (52% reduction). Of note, the effect of Q-922 on AVA and NVA did not statistically differ from that of intravitreal injections of OXT-328 shown in Fig. 1C.

**FIG. 4.**
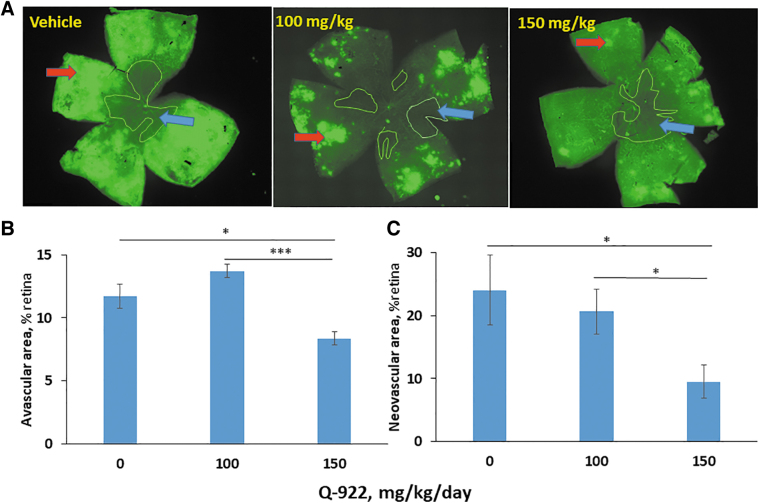
The effect of intraperitoneally administered Q-922 on angiogenesis in OIR. **(A)** Representative images of mouse retinal flat mounts stained with the vascular stain isolectin-B4. Mice were treated with vehicle or one of the indicated doses of Q-922 administered i.p. daily during days P7 and P17. Retinas were harvested on day P17, as in Methods. *Blue and red arrows*: avascular and neovascular areas. The higher Q-922 suppressed both the avascular **(B)** and neovascular **(C)** areas of the retina, compared to vehicle-treated control mice. *n* = 12 eyes/group. Values: mean ± SEM. *P < 0.01; ***P < 0.001.

In other studies, Q-922 was administered intraperitoneally once daily at 2 different doses, 100 and 150 mg/kg body weight, between P7 and P17. As shown in Fig. 4A, representative micrographs of retinal mounts show that both doses decrease AVA and NVA. However, as shown in Fig. 4B and C, quantitative measurements show that the 100 mg/kg dose had no significant effect on either AVA or NVA. In contrast, the higher dose, 150 mg/kg of Q-922, compared to vehicle significantly suppressed the AVA to 8.4% ± 0.5% versus vehicle = 11.7% ± 1.0% (28% reduction), and the NVA to 9.5% ± 2.6% versus vehicle = 24.1% ± 5.6% (61% reduction).

### The effect of CL-717 on OIR and its PK/biodistribution

**FIG. 5.**
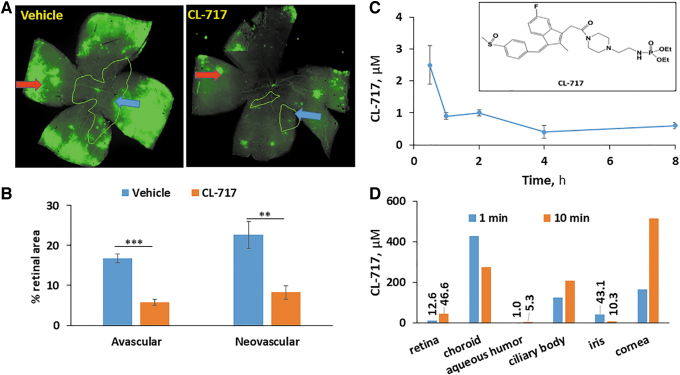
The effect of CL-717 topically applied to the ocular surface on angiogenesis in OIR, and the ocular PK and biodistribution in mice and explanted human eyes. **(A)** Representative images of mouse retinal flat mounts stained with the vascular stain isolectin-B4. Mice were treated with vehicle or CL-717 10% administered as eye drops to the ocular surface 5 × /day during days P12 and P17. Retinas were harvested on day P17, as in Methods. *Blue and red arrows*: avascular and neovascular areas. **(B)** CL-717 suppressed both the avascular and NVAs of the retina, compared to vehicle-treated control mice. *n* = 12–16 eyes/group. Values: mean ± SEM. **P < 0.01; ***P < 0.001. **(C)** Shows the levels of CL-717 in retina of mice following the application of 3 eye drops of CL-717 10% on the ocular surface of adult mice, as in Methods. C_max_ = 2.5 μM; T_max_ = 30 min; and t_1/2_ = 4.7 h. Values: mean ± SEM; *n* = 5. The inset is the molecular structure of CL-717, a modified form of sulindac. **(D)** The levels of CL-717 in explanted human eyes (*n* = 2) measured in various ocular tissues, 1 h after the ocular surface was dipped in a 10% solution of CL-717 for either 1 or 10 min.

Finally, we evaluated the efficacy of CL-717 on OIR. CL-717 10% was applied onto the ocular surface as a single 5-μL eye drop 5 × /day, between 9 am and 5 pm, roughly every 2 h, on days P12-P17 (Table 1). As shown in representative retinal mounts (Fig. 5A) and from quantitative measurements in Fig. 5B, at sacrifice (P17), compared to vehicle control, CL-717 eye drops significantly decreased the AVA from 16.8% ± 1.1% to 5.8% ± 0.8% (65% reduction), and the NVA from 22.7% ± 3.4% to 8.3% ± 1.6% (63% reduction).

The efficacy of CL-717 applied topically to the ocular surface prompted us to assess its retina levels. Indeed, CL-717 reached the retina rapidly, with the following PK parameters: C_max_ = 2.5 μM; T_max_ = 30 min; and t_1/2_ = 4.7 h (Fig. 5C).

The apparent clinical potential of this finding for the control of OIR with a noninjectable agent prompted us to examine its biodistribution in human tissues. Initially, we determined the uptake and distribution of CL-717 in 2 explanted human eyes. These eyes, kept on ice, were studied within 2 h after they were harvested from the donors, a time period without significant tissue degradation.

The ocular surface of 1 eye was exposed to CL-717 (10% aqueous solution) for 1 min and of the other eye for 10 min and both were incubated at 35.5°C (the normal temperature of eyes in humans) for 1 h. CL-717 levels were determined in ocular tissues harvested at 1 h. CL-717 rapidly reached the retina, achieving levels of 12.6 and 46.6 μM after the 1- and 10-min exposure, respectively. The CL-717 concentrations in the choroid, ciliary body, and the cornea ranged between 166.3 and 512.6 μM, while lower levels where found in the aqueous humor and iris (Fig. 5D).

### Safety

No side effect was noted with any of the 3 compounds during their administration in mice as described above, except with topical Q-922. In the formulation we used, Q-922 applied on the ocular surface caused erythema and edema restricted to the cornea and conjunctiva, with the eyelids unaffected. Both resolved within 48 h after discontinuing its administration.

### Q922, CL-717, and OXT-328 suppress angiogenesis in the CAM angiogenesis model

To explore the strong antiangiogenic effect of these 3 compounds in the retina, we employed the CAM model. Because of its rapid vascular growth, this is the model of choice to evaluate the effect of many compounds on growing vessels, including small molecules.^25^

**FIG. 6.**
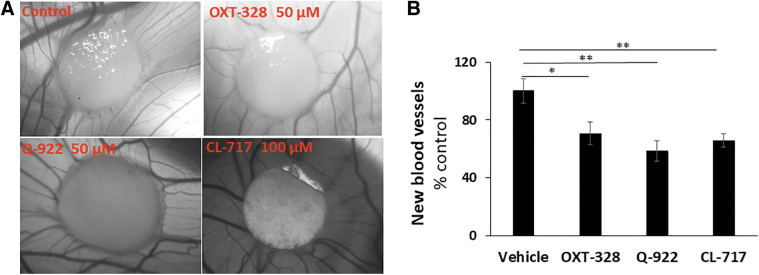
The effect of our test compounds on angiogenesis in the CAM model. **(A)** Representative images of chick embryonic CAM taken after treatment for 72 h with OXT-328 50 μM, Q-922 50 μM, CL-717 100 μM, or vehicle as indicated. **(B)** All 3 compounds suppressed the number of new vessels determined at 72 h and expressed as % control (vehicle-treated). *n* = 5. Values: mean ± SEM. *P < 0.05, **P < 0.01. CAM, chorioallantoic membrane.

As shown in Fig. 6A, and B, all 3 compounds reduced the number of new blood vessels compared with the control: OXT-328 50 μM reduced the average new vessel formation by 29.3%, Q-922 50 μM by 41.4%, and CL-717 100 μM by 34.2%. A significant difference between the effects of these 3 drugs was not observed, even though CL-717 was at a higher dose than either OXT-328 or Q-922.

## Discussion

The control of DR with small molecules remains a major therapeutic challenge, given its prevalence and clinical consequences when not successfully treated. Modified NSAIDs appear to be highly promising pharmacological compounds with a useful biologically profile. These studies represent our efforts to improve on their chemistry and pharmacology and to explore their application to ROP and other proliferative retinopathies.

The 3 compounds studied here belong to the evolving class provisionally known as modified NSAIDs.^26^ OXT-328 has considerably higher anticancer and anti-inflammatory efficacy and safety, compared to sulindac from which it is derived,^12,27–29^ but limited systemic bioavailability due to its susceptibility to hydrolysis by the ubiquitous carboxylesterases.^16^ Q-922 and CL-717 were primarily designed to overcome this limitation by making rational structural changes. For example, CL-717, also based on sulindac, has a different spacer moiety and an amide bond to diethylphosphate, while Q-922 is based on an extensively modified aspirin moiety and has no carboxylic ester or other easily hydrolyzable bonds. We also obtained salts of both. Our PK study confirms their resistance to hydrolases.

The synthesis of Q-922 and CL-717 was achieved by no more than 4-step processes, yielding final products with purities approaching 100%. Overall, these methods were robust and easily reproducible with over 80% yields in most steps (see Supplementary Data S1).

The mouse OIR model that we used in these studies is highly relevant to DR.^30–32^ OIR faithfully recapitulates abnormal blood vessel growth in the retina, the hallmark of such retinal diseases as ROP, proliferative DR, and the wet form of age-related macular degeneration. In fact, OIR has been widely employed not only to decipher the mechanism of retinal angiogenesis but also in the development of widely used injectable anti-VEGF agents.^7,33^

OXT-328 has already been evaluated in ocular therapeutics generating excellent results in the treatment of dry eye disease in rabbit models.^34^ Interestingly, OXT-328 maintains its structural integrity in the rabbit cornea and conjunctiva for several hours, being metabolized at a relatively slow rate^24^; the carboxylesterase profile of the rabbit ocular surface, similar to the human, is drastically different from that of the mouse.^35^

The latter explains why the topical application of OXT-328 as eye drops to mice was not efficacious in OIR. The intravitreal injection of OXT-328, however, did inhibit retinal angiogenesis. This effect was robust and included both aspects of the response of the retina to the hyperoxic conditions, including the processes of avascularization and neovascularization. What apparently underlies this strong efficacy is the fact that OXT-328 survives largely intact when injected into the vitreous, with only a minimal amount of this compound being metabolized since the retinal carboxylesterase activity is much lower than the cornea.^35,36^

Q-922 administered either intravitreally or systemically (i.p.) was efficacious in suppressing retinal angiogenesis in mice. The effect of the former was more pronounced compared to the latter. The intraperitoneal administration of Q-922 was efficacious, as expected of a nonhydrolyzable compound that escapes the action of esterases in the systemic circulation, the liver, and the eye itself. It is conceivable that the irritation of the ocular surface in response to its topical administration could be prevented by a different formulation of Q-922.

CL-717, unique among these 3 compounds, produced a remarkable effect on retinal angiogenesis when applied topically as eye drops. This effect was quantitatively stronger compared with the other 2 compounds, which needed to be injected into the vitreous. Our mouse PK observations are consistent with the therapeutic effect of CL-717. While the final pH of the CL-717 eye drops was 5.0, a value that might cause some discomfort if administered to humans, it may also affect the bioavailability of this experimental compound. Of note, approved ocular solutions have pH values below 5.^37^

Interestingly, our observations with 2 explanted human eyes, even though limited, lend credence to the speculation that CL-717 given as eye drops may traverse the human eye and reach the retina at levels that suppress angiogenesis.

Supportive evidence of a direct antiangiogenic effect of these compounds comes from the studies with chicken CAM, an informative and widely used model of angiogenesis. All 3 compounds suppressed angiogenesis in CAM, consistent with our results from the OIR model.

Our results compare and contrast with results obtained with intravitreal injection of anti-VEGF agents using the same murine OIR model.^33^ For example, in one study, intravitreal anti-VEGF treatment caused about 70% reduction of NVA in the retina, but increased 1–2-fold the AVA. In fact, our 3 compounds administered topically or systemically decreased both the AVA and NVA.

At this time, we can only speculate as to the mechanism of antiangiogenic action of our modified NSAIDs. Most likely, the effect is different from other anti-VEGF agents. If we were to assume that the initial hyperoxia increases the presence of free oxygen radicals and that this leads to an antiangiogenic effect on developing blood vessels in the retina^38,39^ and the suppression of VEGF release, subsequent return to room air and the initial relative hypoxic conditions provide a powerful stimulus for the release of VEGF peptides and angiogenesis. Since we have shown that our modified NSAIDs inhibit this neovascularization, we speculate that they also increase the “oxidative stress” of either or both of those cells that release VEGF or are stimulated by VEGF to form new blood vessels in the retina.

Currently, there is keen interest in the development of small molecules, which can be delivered by either eye drops or systemically for the treatment of proliferative retinopathies.^19,40^ Our compounds appear to hold promise for such applications. If clinically successful, they will greatly simplify the treatment of these diseases, especially in view of their ease of delivery. Further work will be required to assess this possibility.

## Conclusions

These studies demonstrate a broad antiangiogenic effect of structurally distinct modified NSAIDs when administered through 3 different routes of administration. Of them, the topical application of CL-717 as eye drops is clinically the most appealing as it raises the possibility of treating proliferative retinopathies, including DR, without intraocular injections of OXT-328 or Q-922 injected either intraocularly or intraperitoneally. Thus, we believe these 3 compounds merit further development to explore their therapeutic potential in the treatment of proliferative retinopathies, a major cause of vision loss worldwide.

## Supplementary Material

Supplemental data
