# Supplementary material for: The Antiangiogenic Effect and Ocular Pharmacology of Novel Modified Nonsteroidal Anti-Inflammatory Drugs in the Treatment of Oxygen-Induced Retinopathy
Source: J Ocul Pharmacol Ther. 2023 May 10;39(4):279–89. doi: 10.1089/jop.2022.0113 (PMC10178932; doi:10.1089/jop.2022.0113)
Supplement: Supplemental data [file Supp_Data.docx]

**Supplemental Data: *Ocular pharmacology and anti-angiogenic effect of novel modified NSAIDs in the treatment of oxygen-induced retinopathy***

Authors: Wei Huang , Liqun Huang, Ziyi Wen, Robert A. Honkanen, Basil Rigas

**Synthesis of test compounds**

The synthesis of Q-922 and CL-717 is described below. The synthesis of OXT-328 has already been reported (Anti-inflammatory compounds and uses thereof - Patent US-8236820-B2).

The structure of all three compounds are shown in Supplemental Fig 1.

**
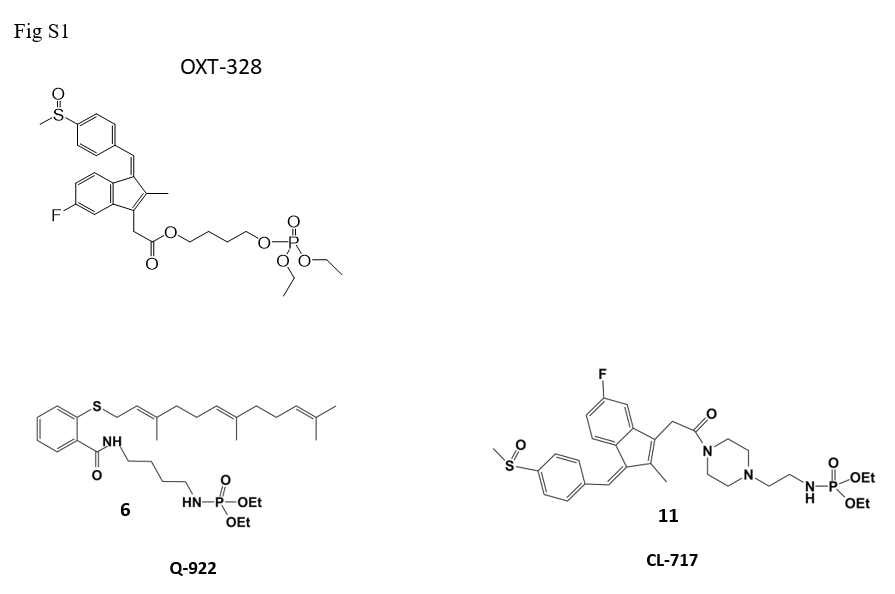
**

**Supplemental Fig 1. The chemical structures of novel NSAIDs with anti-angiogenic properties.** The synthesis of OXT-328 or phospho-sulindac has been reported elsewhere. Q-922 is a heavily modified form of aspirin and CL-717 is a modified form of sulindac. In both molecules the carboxylic ester of phosphor-sulindac has been replaced by a non-hydrolyzable bond making these NSAIDs resistant to hydrolysis by endogenous carboxylesterases.

**Synthesis of Q-922 (Compound 6)**

The synthesis of Q-922, is depicted in Supplemental Fig 2, proceeds in three steps, namely the synthesis of compounds **3**, **4** and **6**.

**Synthesis of compound 3 (tert-butyl 4-(2-((2E,6E)-3,7,11-trimethyldodeca-2,6,10-trienyl- thio)benzamido) butylcarbamate)**

To a mixture of compound (**1**) (5.00 g, 13.95 mmol) and HOBt (1.89 g, 13.95 mmol, 1.0 eq.) in THF (60.0 mL) in an ice bath was added DCC (3.45 g, 16.74 mmol, 1.2 eq.). The resulting slurry was stirred at room temperature for 2 h. Compound (**2**) (3.15 g, 16.74 mmol, 1.2 eq.) in THF (30.0 mL) was added to this slurry. The reaction mixture was stirred at room temperature overnight, concentrated, then diluted with EtOAc (200 mL), filtered, washed with EtOAc (20 mL × 3). The combined organic layer was treated with NaHCO_3_ (40.0 mL), NaOH (40 mL, 2.0 N), brine (40 mL × 2), dried over MgSO_4_, evaporated, and purified by flash chromatography (0% -30% EtOAc/heptane) to afford compound (**3**) (6.51 g, 88.2%).

**
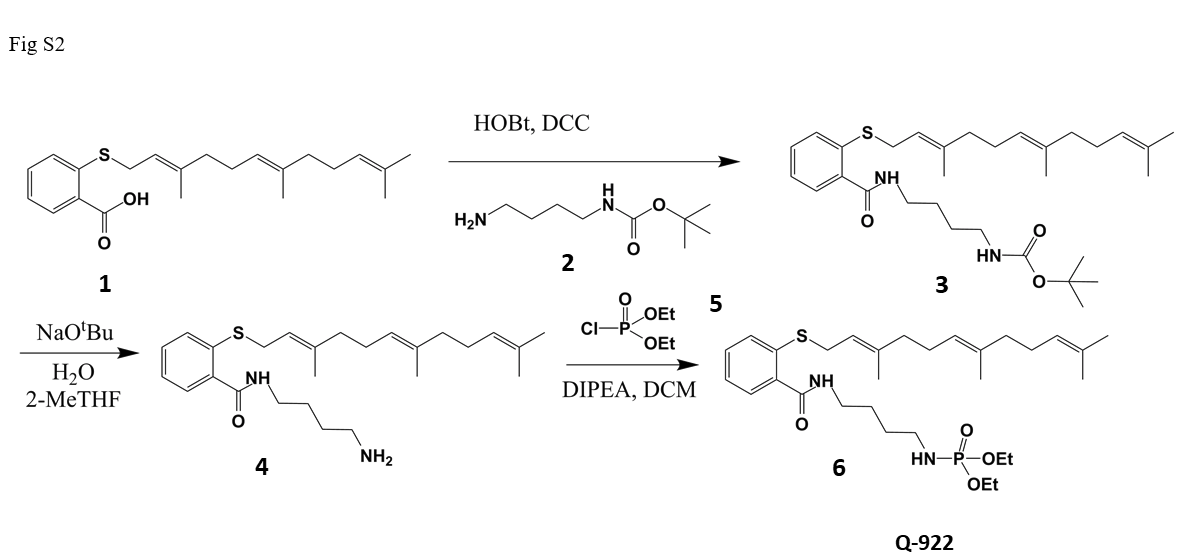
**

**Supplemental Fig 2. Synthesis of Q-922.** The synthesis was carried out as in Materials and Methods. Briefly, (1) was coupled with (2) to obtain (3), which yielded (4), which was then reacted with diethylchlorophosphate to yield Q-922 (6)

**Synthesis of compound 4 (N-(4-aminobutyl)-2-((2E,6E)-3,7,11-trimethyldodeca-2,6,10-trieny- lthio)benzamide)**

A suspension of compound (**3**) (3.86 g, 7.3 mmol), NaO*t*Bu (6.92 g, 72.0 mmol, 10 eq.), H_2_O (0.17 mL, 1.0 eq.) in 2-MeTHF (800 mL) was heated to refluxed for 15 h. After the reaction was complete, the mixture was cooled in an ice bath, quenched with saturated ammonium chloride (50.0 mL) to adjust pH to 10-11. The aqueous layer was extracted with EtOAc (40.0 mL x 4), washed with H_2_O (40.0 mL × 2), brine (30.0 mL), dried over MgSO_4_, evaporated, and purified by flash chromatography (5% -50% MeOH/DCM) to afford compound (**4**) (2.64 g) in 84.3% yield.

**Synthesis of compound 6 (diethyl 4-(2-((2E,6E)-3,7,11-trimethyldodeca-2,6,10-trienylthio) benzamido)butylphosphoramidate)**

To a solution of compound (**4**) (4.73 g, 11.04 mmol), DIPEA (5.80 mL, 3 eq.) in DCM (80 mL) was added compound (**5**) (1.92 mL, 1.2 eq.) in an ice bath. The resulting mixture was stirred at room temperature for 2 h. The mixture was evaporated, diluted with EtOAc (150 mL), washed with NaHCO_3_ (40.0 mL), H_2_O (40.0 mL × 3), brine (30.0 mL), dried over MgSO_4_, evaporated, purified by different solvent systems (0% - 5% MeOH/DCM) and (50% - 100% EtOAc/heptane) to give product **6** (5.05 g) as a light-yellow oil in 83.7% yield. Fig. S3A-B illustrates the ^1^H NMR spectrum of compound **6** and its HPLC chromatogram (single peak).

**Synthesis of CL-717 (Compound 11)**

The synthesis of CL-717, depicted in Supplemental Fig 3, proceeds in four steps, namely the synthesis of compounds **9**, **10, 11** and the salt of compound **11.**

**
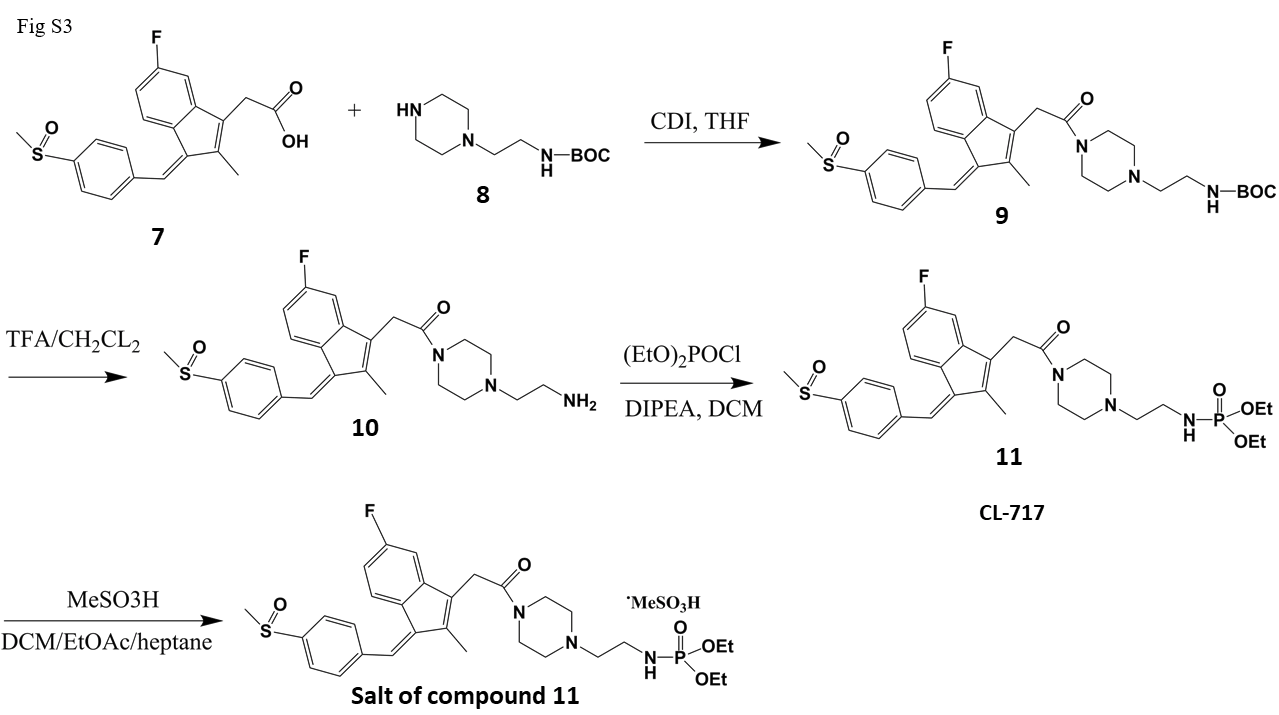
**

**Supplemental Fig 3. Synthesis of CL-717**. The synthesis was carried out as in Materials and Methods. Briefly, (**7**) was reacted with (**8**) according to the coupling method to obtain (**9**), which in the presence of TFA/CH_2_Cl_2_ yielded (**10**), which was then reacted with diethylchlorophosphate to yield CL-717 (**11**)

**Synthesis of intermediate 9 ((Z)-diethyl 2-(4-(2-(5-fluoro-2-methyl-1-(4-(methylsulfinyl) benzylidene)-1H-inden-3-yl)acetyl)piperazin-1-yl)ethylphosphoramidate)**

A mixture of sulindac (9.36 g, 26.2 mmol) and CDI (4.92 g, 30.1 mmol, 1.15 eq.) in THF (150 mL), was stirred at room temperature for 1-2 h to generate a thick slurry. After the complete consumption of sulindac, compound (**8**) (7.83 g, 34.1 mmol, 1.30 eq.) was added to the slurry. The resulting mixture was stirred at room temperature overnight to give a clear yellowish solution. After the reaction was complete as monitored by HPLC, the solvent was evaporated under reduced pressure at room temperature. EtOAc (200 mL) and H_2_O (60.0 mL) were then added to the reaction mixture. The organic layer was washed with H_2_O (40.0 mL × 3), brine (30.0 mL), dried over MgSO_4_, and evaporated to give intermediate (**9**) (17.2 g). The material was used in the next step without further purification.

**Synthesis of intermediate 10 (Z)-1-(4-(2-aminoethyl)piperazin-1-yl)-2-(5-fluoro-2-methyl-1-(4-methylsulfinyl)benzylidene)-1H-inden-3-yl)ethanone**

To a solution of intermediate (**9**) (17.2 g) in DCM (90.0 mL) was added TFA (35.0 mL) in ice bath. The resulting solution was stirred overnight. After the reaction was complete as monitored by HPLC, the reaction mixture was evaporated under reduced pressure at room temperature to give intermediate (**10**) (40.6 g with excess TFA residue). The material was used in the next step without further purification

**Synthesis of compound 11 ((Z)-diethyl 2-(4-(2-(5-fluoro-2-methyl-1-(4-(methylsulfinyl) benzylidene)-1H-inden-3-yl)acetyl)piperazin-1-yl)ethylphosphoramidate)**

To a solution of intermediate (**10**) (40.7 g with excess TFA, about 26 mmol) in DCM (200 mL) in the presence of DIPEA (150 mL) in ice bath was added diethyl chlorophosphate (6.7 g, 1.5 eq.) in DCM (20 mL) over 4 h via a syringe pump. After the reaction was complete as monitored by HPLC, the reaction mixture was concentrated, then diluted with EtOAc (200 mL), washed with NaHCO_3_ (50 mL × 2), H_2_O (40 mL × 4), brine (50 mL × 2), and evaporated and purified by flash chromatography (0% - 5% MeOH/DCM) to afford compound **11** (6.56 g) (final yield: 41.8%).

**Synthesis of the salt of compound 11**

A solution of compound **11** (8.41 g) in DCM (100 mL) was stirred at 0 °C, then methanesulfonic acid (1.32 g, 1.0 eq.) in DCM (10.0 mL) was added slowly. EtOAc (100 mL) was added to the above mixture very slowly followed by heptane (400 mL). The resulting suspension was stirred for 2 h, filtered, washed with heptane (30.0 mL x 3) to give yellow solid. This yellow solid was dried under high vacuum, then dissolved in H_2_O (50 mL), freeze drying to furnish compound **11** salt (9.40 g).

**HPLC**

The 1HNMR and HPLC chromatograms of the experimental compounds, Q-922 and CL-717 are shown in Supplemental Fig. 4. The HPLC system consisted of a Waters Alliance 2695 Separations Module (Milford, MA, USA) equipped with a Waters 2998. Column: For OXT-328 and CL-717 Thermo Hypersil BDS C18 column (150 x 4.6 mm, particle size 3 mm); for Q-922 Halo® C18 column (150 x 4.6 mm, particle size 2.7 mm). Elution system: For OXT-328, the mobile phase consisted of a gradient between solvent A [formic acid, H_2_O (0.1:99.95 v/v)] and solvent B (CH_3_CN) at a flow rate of 1 mL/min at 30°C. Elution time: 10 min. For Q922 and CL-717, the mobile phase consisted of a gradient between solvent A [trifluoroacetic acid, H_2_O (0.05:99.95 v/v)] and solvent B [trifluoroacetic acid, CH_3_CN (0.05:99.95 v/v)] at a flow rate of 1 mL/min at 30°C. Elution time: 15 minutes. Detection wavelength: 328 nm for OXT-328; 260 nm for Q-922; and 328 nm for CL-717. Both Q-922 and CL-717 could be eluted by HPLC as single peaks.

**Synthesis of Q-922 and CL-717**

Q-922 and CL-717 synthesized, as shown above in Supplemental Figs. 2-3, were characterized by ^1^H-NMR and HPLC (Supplemental Fig. 4). The HPLC chromatograms showed a single peak for each compound at 6.87 and 6.81 min, respectively. The yield of the reactions was excellent, and the production of salt forms makes these two compounds water-soluble.

**
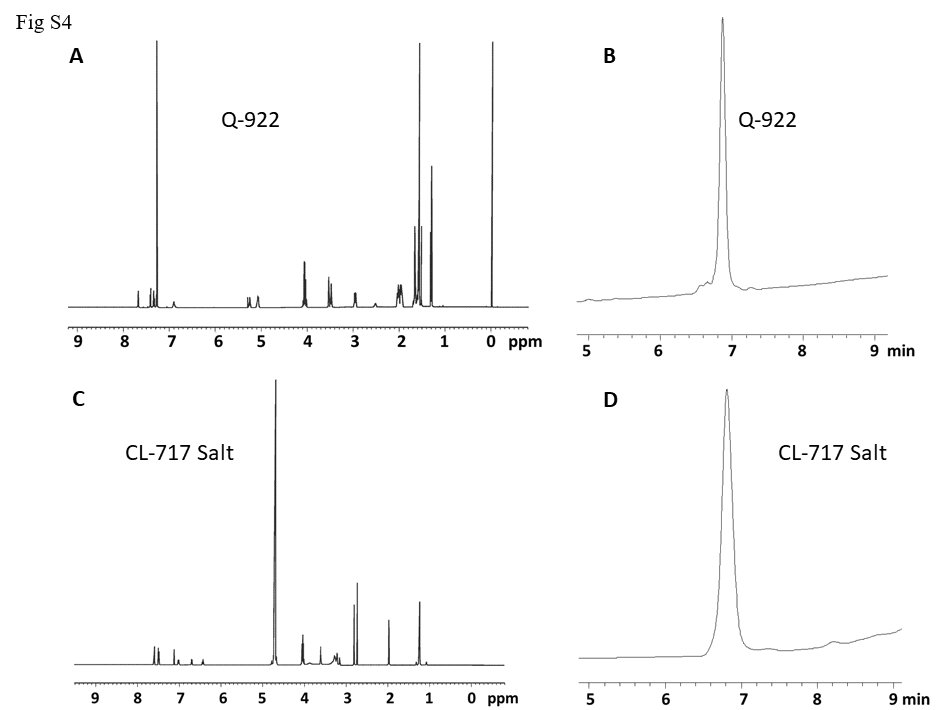
**

**Supplemental Fig 4. ^1^HNMR and HPLC of Q-922 and CL-717.** The ^1^HNMR and HPLC chromatogram of Q-922 (A and B, respectively) and CL-717 (C and D) obtained as in Methods. Both Q-922 and CL-717 appear as single peaks in their respective HPLC chromatograms.
